# Prevalence of poor glycemic control in diabetic hypertensive patients: a systematic review and meta-analysis

**DOI:** 10.1186/s12902-026-02357-0

**Published:** 2026-06-15

**Authors:** Dula Dessalegn Bosho, Yonas Deressa Guracho, Selamawit Worku Asmare, Behailu Terefe Tesfaye, Diriba Dereje Olana, Tesema Etefa Birhanu

**Affiliations:** 1 Institute of Health, School of Pharmacy, Clinical Pharmacy, Jimma University, Jimma, Ethiopia; 2 Faculty of Engineering and Information Sciences, University of Wollongong, Wollongong, Australia; 3 Department of Dermatology and Venereology, Yekatit-12 Hospital Medical College, Addis Ababa, Ethiopia; 4 Institute of Health, Department of Biomedical Science (Medical Physiology), Jimma University, Jimma, Ethiopia; 5 Institute of Health, Department of Biomedical Science (Clinical Anatomy), Jimma University, Jimma, Ethiopia; 6 College of Medical and Health Sciences, Bahir Dar University, Bahir Dar, Ethiopia

**Keywords:** Prevalence, Glycemic control, Diabetes mellitus, Hypertension, Meta-analysis

## Abstract

**Introduction:**

Poor glycemic control is a significant public health issue, contributing to the development of complications, increasing healthcare costs, and quality of life. The objective of the meta-analysis is to estimate the prevalence of poor glycemic control and its prevalence according to diagnostic criteria in diabetic hypertensive patients. Findings will be critical in reducing the burden of poor glycemic control and complications.

**Methods:**

A systematic review and meta-analysis were conducted in accordance with the PRISMA 2020 guidelines. Three electronic databases (PubMed, Web of Science, and SCOPUS) were searched for studies published in English between January 2015 and January 2025. Primary studies assessed glycemic control among diabetic-hypertensive patients using one or more of the following: a fasting blood glucose (FBG) level (> 126 mg/dL, > 130 mg/dL, or > 130 mg/dL) and/or glycated hemoglobin A1C level (≥ 7%). Random-effects models (metaprop, REML) were used to estimate pooled prevalence. Heterogeneity, sensitivity test, subgroup analysis, and publication bias assessments were performed.

**Results:**

In this study, ten papers involving a total of 2552 individuals were utilized to derive the pooled estimate. The pooled prevalence of poor glycemic control was 59% (95%CI; 0.49–0.69). Based on diagnostic criteria the pooled prevalence of poor glycemic control in diabetic hypertensive patients was 46% (95%CI: 0.31–0.61), and 65% (95%CI; 0.54–0.76) for fasting blood glucose and glycosylated hemoglobin respectively. There is substantial heterogeneity but no significant publication bias on visual inspection of the forest plot.

**Conclusion:**

Around two-third of study participants have poor glycemic control in diabetic hypertensive patients. This finding is important as a baseline for refining clinical guidelines and improving long-term outcomes for this high-risk group. Future researchers will evaluate effective interventions to improve glycemic control in diabetic hypertensive patients across diverse study settings.

**Clinical trial number:**

Not applicable.

**Supplementary Information:**

The online version contains supplementary material available at 10.1186/s12902-026-02357-0.

## Background

Cardiovascular diseases (CVDs) are the leading cause of death globally, representing 32% of all global deaths. More than 75% of deaths from CVD occur in low- and middle-income nations [1]. Diabetes mellitus (DM) is a prevalent and severe chronic disease that poses a significant global public health concern [2, 3]. DM affects 537 million people worldwide (1 in 10 adults), whereas 24 million adults in Africa (1 in 22 adults) have the disease. This figure is expected to increase to 783 million worldwide and 55 million in Africa by 2045 [4]. Approximately 1.28 billion persons between the ages of 30 and 79 globally have hypertension, with the majority (two-thirds) residing in low- and middle-income nations [5, 6].

Hypertension is the most common comorbidity of diabetes; more than two-thirds of people with type 2 diabetes have hypertension [7]. Hypertension may account for up to 75% of CVD in diabetes, necessitating aggressive treatment in individuals with coexistent diabetes and hypertension [8]. Both diabetes and hypertension patients have twice the risk of CVDs (stroke and heart attack) than non-diabetic hypertensive patients [9]. Diabetic hypertensive patients are also at increased risk for complications, including retinopathy and kidney disease. The risk of diabetic complications and CVD morbidity, and mortality was attributed to poorly glycemic control [10–12]. Anthropometric indicators, such as body mass index and waist-to-height ratio, serve as significant predictors of adverse cardiovascular outcomes in individuals with type 2 DM. Their role in contributing to poor glycemic control and heightened cardiometabolic risk has been well documented [13].

According to studies, only about 50% of diabetics worldwide have good glycemic control [14], while a prior meta-analysis revealed that the majority of diabetic patients had poor glycemic control [15, 16]. According to estimates, 3.7 million people died from high blood glucose in 2012 (1.5 million from diabetes, and an additional 2.2 million from CVD and chronic renal disease). Approximately 7% of deaths worldwide are attributed to excessive blood glucose in men and 8% in women aged 20 to 69 years [17]. Poor glycemic control is a significant public health issue, contributing to the development of diabetes-related complications, increasing healthcare costs, and reducing life expectancy and quality of life [18]. Poor glycemic control has been identified as a global growing problem for patients with diabetes, with over 60% of patients failing to meet the desired glycemic target, despite the evidence that strict glycemic control and application of digital health intervention have significantly improved patient health outcomes and reduced complications of diabetes [19, 20].

Despite the expanding body of literature regarding glycemic management in individuals with diabetes mellitus (DM), a significant gap persists in the understanding of the burden associated with poor glycemic control, particularly among patients with DM who also have comorbid hypertension. And numerous studies have reported the prevalence of glycemic control in diabetic hypertensive patients with wide variable ranges. This highlights the inconsistency of findings across settings. The findings of the study provide input for the clinical implications (burden and risk factors) of care for patients with diabetes and hypertension. No prior systematic review has synthesized data on the prevalence of poor glycemic control exclusively within this high-risk group. So, the objective of the study is to estimate the pooled prevalence of poor glycemic control among diabetic hypertensive patients. Therefore, conducting independent pooled analysis is crucial to determine how common poor glycemic control is in this high-risk group and to guide targeted clinical and policy strategies.

## Methods

### Data sources and search strategies

A comprehensive electronic-based literature search was conducted following the Preferred Reporting Items for Systematic Reviews and Meta-Analyses (PRISMA) guidelines [21]. We registered this meta-analysis with PROSPERO (CRD: 42024612181) to avoid unnecessary replication. We searched the PubMed, Web of Science, and SCOPUS electronic databases using a comprehensive search strategy with Boolean operators (“OR” and “AND”). These are keywords we used for database search: “Poor OR glycemic OR control OR blood sugar OR A1C AND diabetes mellitus co-morbid hypertension OR diabetes mellitus hypertension”. All database search results are presented below in the text box.

**Table:** 

((((((Poor) OR (glycemic)) OR (control)) OR (blood sugar)) OR (A1C)) AND (diabetes mellitus co-morbid hypertension)) OR (diabetes mellitus hypertension). For PubMed
(Poor AND glycemic AND control AND diabetes AND mellitus OR diabetic OR hypertension OR hypertensive OR diabetes AND mellitus AND co-morbid AND hypertension AND patients OR individuals). For SCOPUS
Poor (Topic) OR glycemic (Topic) OR control (Topic) OR blood sugar (Topic) OR A1C (Topic) AND diabetes mellitus co-morbid hypertension (Topic) OR diabetes mellitus hypertension. For WOS

### Selection of studies

**Fig. 1:**
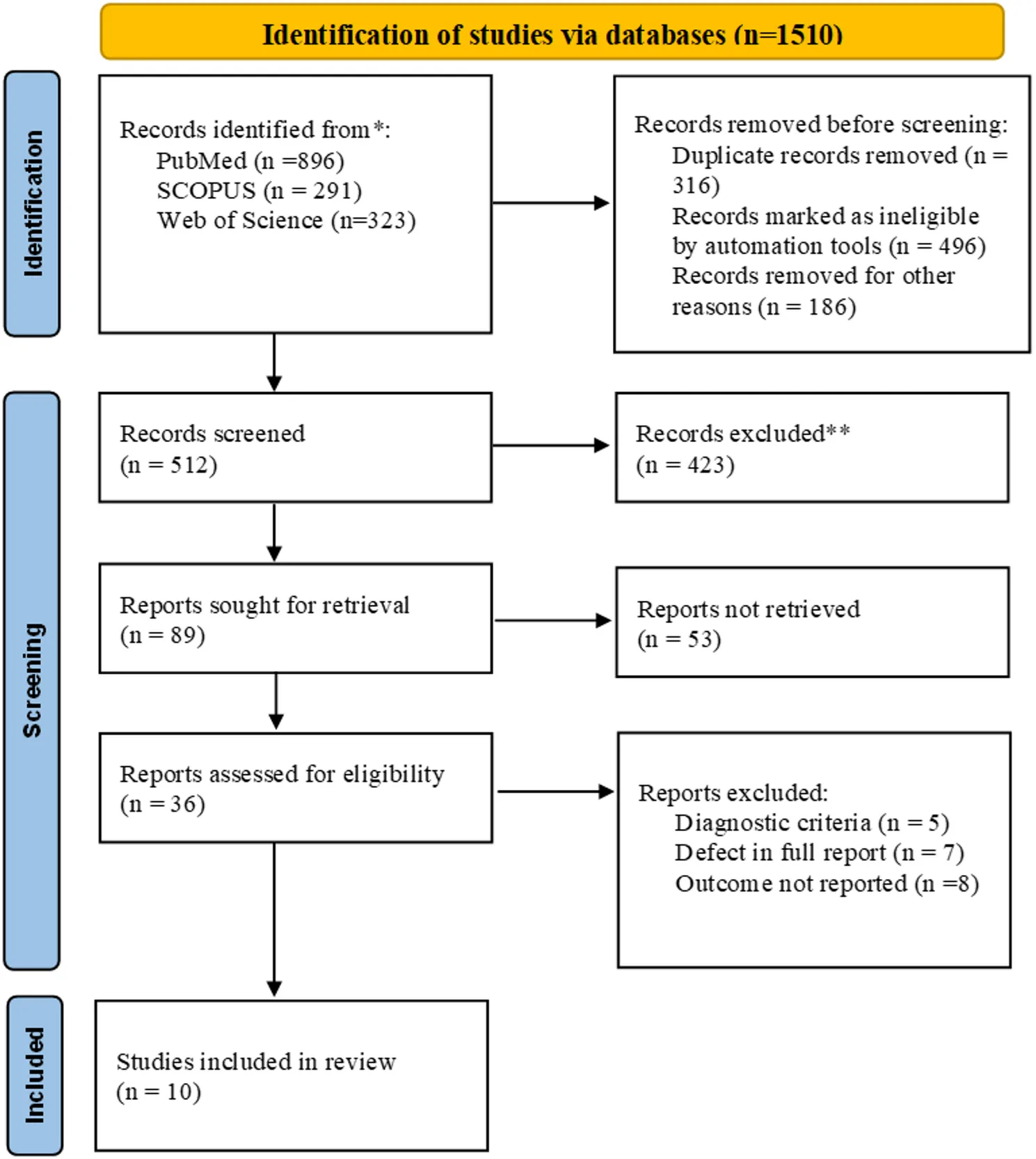
PRISMA flow diagram of article selection for systematic review and meta-analysis of poorly controlled glycemia in diabetic hypertensive patients [21]

All selected articles were imported to Zotero online. The duplicate articles are removed using the Zotero duplication check and manager. A thorough screening method identified relevant studies that met the inclusion criteria, and the research goals were incorporated. If there were disagreements, the writers collaborated to address them through careful consideration and dialogue. Two of the reviewers (DDB and DDO) screened the titles and abstracts of each article to identify potentially eligible studies. Then, (TE and YD) independently extracted relevant data from full-length articles that fulfilled the inclusion criteria (Fig. 1). Discrepancies were resolved by mutual consent following discussion and independent review by the authors (TE, SW, and BT).

### Eligibility criteria

The target population includes adults diagnosed with diabetic-hypertensive conditions or DM co-morbid hypertension.

Primary studies were included if they met the following criteria:

- Population: adults diagnosed with diabetic-hypertensive conditions.
- Study design: quantitative study or cross-sectional study.
- Outcomes: studies that assessed glycemic control using one or more of the following: a fasting blood glucose (FBG) level (of > 126 mg/dl, > 130 mg/dl, or > 130 mg/dl) and/or a glycated hemoglobin A1C level (of ≥ 7%).
- Language: English.
- Publication period: 2015–2025.

### Exclusion criteria

- Studies that did not specify the criteria for assessing good or poor glycemic control.
- Studies that didn’t report the number of patients with good or poor glycemic control.
- Unpublished article, case reports, conference papers, or dissertations.

### Outcome measurement

Patients with an FBG level of ≥ 126 mg/dl or ≥ 130 mg/dl, or patients with an A1C level of ≥ 7%, were considered to have poor glycemic control [22]. The proportions of poor glycemic control were calculated from the total number of DM-hypertensive patients examined for glycemic control.

### Quality assessment

Quality assessment was performed using the nine items. Two authors independently evaluated the quality of the selected publications using the Joanna Briggs Institute. Meta-analysis of Statistics Assessment and Review Instrument (JBI-MAStARI) critical assessment technique, and the third author resolved any disagreements [23]. This strategy was used to collect primary outcome data and evaluate the methodological rigor and quality of the included studies.

### Data extraction

All authors systematically extracted relevant articles using a prepared form and finally put the extracted data in a Microsoft Excel spreadsheet. The name of the authors, the year of publication, the study location or country, the study setting, the age (mean age), sex ratio, adherence ratio, the study population, the sample size, prevalence, the response rate, and diagnostic criteria were all included in the data extraction. Data regarding clinical and laboratory characteristics (FBG and A1C) were extracted. This methodical approach to data extraction ensured that all relevant information from the included studies was systematically collected and organized, facilitating assessment of the research results.

### Data synthesis and statistical analysis

Meta-analysis software, STATA version 18 [24], was used to perform meta-analyses of the proportions of poor glycemic control among diabetic-hypertensive patients based on diagnostic criteria. To simplify the analysis, the pertinent results were transformed into an event/count format and then imported into STATA version 18 for further analysis. The pooled prevalence was computed using a statistical technique based on the meta-analysis proportion. The I^2^ statistic, which quantifies the degree of variability or heterogeneity among studies in a meta-analysis, was also used to assess heterogeneity in the included studies and to determine the appropriate model. Using this comprehensive technique, the study considers the variances between the studies and offers important information regarding the generalizability and overall consistency of the results. In general, a forest plot was used to present the investigation and visualize the results. Commonly used criteria to denote low, moderate, and high degrees of heterogeneity are 25%, 50%, and 75% of I^2^. Using a visual funnel plot, publication bias was evaluated [25].

## Results

We searched a total of 1510 papers from various electronic databases between November 1, 2024, and December 15, 2024. Among the identified articles, 10 studies with a total sample size of 2552 were eligible for systematic review and meta-analysis (as indicated in Fig. 1). All investigations were conducted in referral and teaching/specialized hospitals, using the same study population and design. The studies included: four studies were undertaken in Ethiopia [26–29], with two each in East Africa [30, 31], West Africa [32, 33], and Asia [19, 34]. The study’s outcomes were assessed using two diagnostic criteria: FBG > 130 mg/dl [26–28] and HbA1c ≥ 7 [19, 29–34]. The individual prevalence of poor glycemic control increased from 34.35% to 85.4%. (Show in Table 1). We assessed the quality of included articles by the JBI Critical Appraisal Tools checklist. This checklist contains nine items, each of which can be answered “Yes,” “No,” or “Not applicable (NA)”. Five of the included studies achieved the highest possible score of 9 out of 9, while the others scored between 7 and 9 (as shown in Table 2).

**Table 1:** Characteristics of the included studies

Author and year	Country	Mean age (in years)	Setting	Population	Sample size	Sex ratio (M/F)	Adherence (adherent/Non-adherent)	Diagnostic criteria	Prevalence	Response rate
Muleta et al., 2017 [26]	Ethiopia	50.69 ± 13.71	JUMC	DM-HTN	131	34/40	48/26	FBG >130 mg/dl	45(34.35%)	NR
Yimam et al., 2020 [27]	Ethiopia	54.4 ± 11.7	JUMC	DM-HTN	309	194/106	143/37	FBG >130 mg/dl	180(60%)	97.1%
Dedefo et al., 2020 [28]	Ethiopia	51.2 ± 12.2	Nekemte Referral Hospital	DM-HTN	186	83/103	NR	FBG >130 mg/dl	80(43%)	NR
Akalu and Belsti., 2020 [29]	Ethiopia	56 ± 12	Debre Tabor General Hospital	DM-HTN	225	225/153	NR	HbA1c ≥ 7	160(71.4%)	100%
Semvua B. K. et al., 2017 [30]	Tanzania	> 18	Bugando Medical Centre	DM-HTN	206	134/161	NR	HbA1c ≥ 7	149(72.35)	100%
Alhassan et al., 2023 [32]	Ghana	57.5 ± 13.2	Adabraka Polyclinic Centre	T2DM-HTN	329	144/185	121/208	HbA1c ≥ 7	141(42.9%)	100%
Adegoke et al., 2022 [33]	Nigeria	61.2 ± 0.7	Tertiary health-facility in Lagos	T2DM-HTN	238	58/97	73/82	HbA1c ≥ 7	137(73.2%)	78.6%
Saasita et al., 2021 [31]	Uganda	> 18	Mbarara regional referral	T2DM-HTN	206	48/122	NR	HbA1c ≥ 7	176(85.4%)	100%
Jarab et al., 2023 [19]	Jordan	62 ± 10	King Abdullah and Royal Medical hospital	T2DM-HTN	522	267/255	NR	HbA1c ≥ 7	268(51.3%)	NR
Burney et al., 2023 [34]	Pakistan	> 18	PRH/IIMC/RIU	T2DM-HTN	200	110/90	NR	HbA1c ≥ 7	113(56.5%)	NR

DM; Diabetes mellitus, FBG; Fasting blood glucose, HbA1c; glycated hemoglobin, JUMC; Jimma University Medical Centre, PRH/IIMC/RIU; Pakistan Railway hospital/ Islamic International Medical College/Riphah International University,T2DM; Type 2 diabetes mellitus, NR; Not reported

**Table 2:** Methodological quality of included studies using the JBI assessment scale

Author and year	Quality of the domain									Total score
	Q1.	Q2.	Q3.	Q4.	Q5.	Q6.	Q7.	Q8.	Q9.	
Muleta et al., 2017 [26]	Y	Y	Y	Y	Y	Y	Y	Y	Y	9/9
Yimam et al., 2020 [27]	Y	Y	Y	Y	Y	Y	Y	Y	Y	9/9
Dedefo et al., 2020 [28]	Y	Y	N	Y	Y	Y	Y	Y	Y	8/9
Akalu and Belsti., 2020 [29]	Y	Y	Y	Y	Y	Y	N	Y	Y	8/9
Semvua B. K. et al., 2017[30]	Y	Y	N	Y	Y	Y	N	Y	Y	7/9
Alhassan et al., 2023[32]	Y	Y	Y	Y	Y	Y	Y	Y	Y	9/9
Adegoke et al., 2022 [33]	Y	Y	N	Y	Y	Y	Y	Y	N	7/9
Saasita et al., 2021[31]	Y	Y	N	Y	Y	Y	N	Y	Y	7/9
Jarab et al., 2023 [19]	Y	Y	Y	Y	Y	Y	Y	Y	Y	9/9
Burney et al., 223 [34]	Y	Y	Y	Y	Y	Y	Y	Y	Y	9/9

**Checklist items used **Q1. Was the sample frame appropriate address the target population? Q2. Were study participants sampled in an appropriate way? Q3. Was the sample size adequate? Q4. Were the study subjects and the setting described in detail? Q5. Was the data analysis conducted with sufficient coverage of the identified sample? Q6. Were valid methods used for the identification of the condition? Q7. Was the condition measured in a standard, reliable way for all participants? Q8. Was there an appropriate statistical analysis? Q9. Was the response rate adequate, and if not, was the low response rate managed appropriately?

### Prevalence of poor glycemic control in diabetic hypertensive patients

**Fig. 2:**
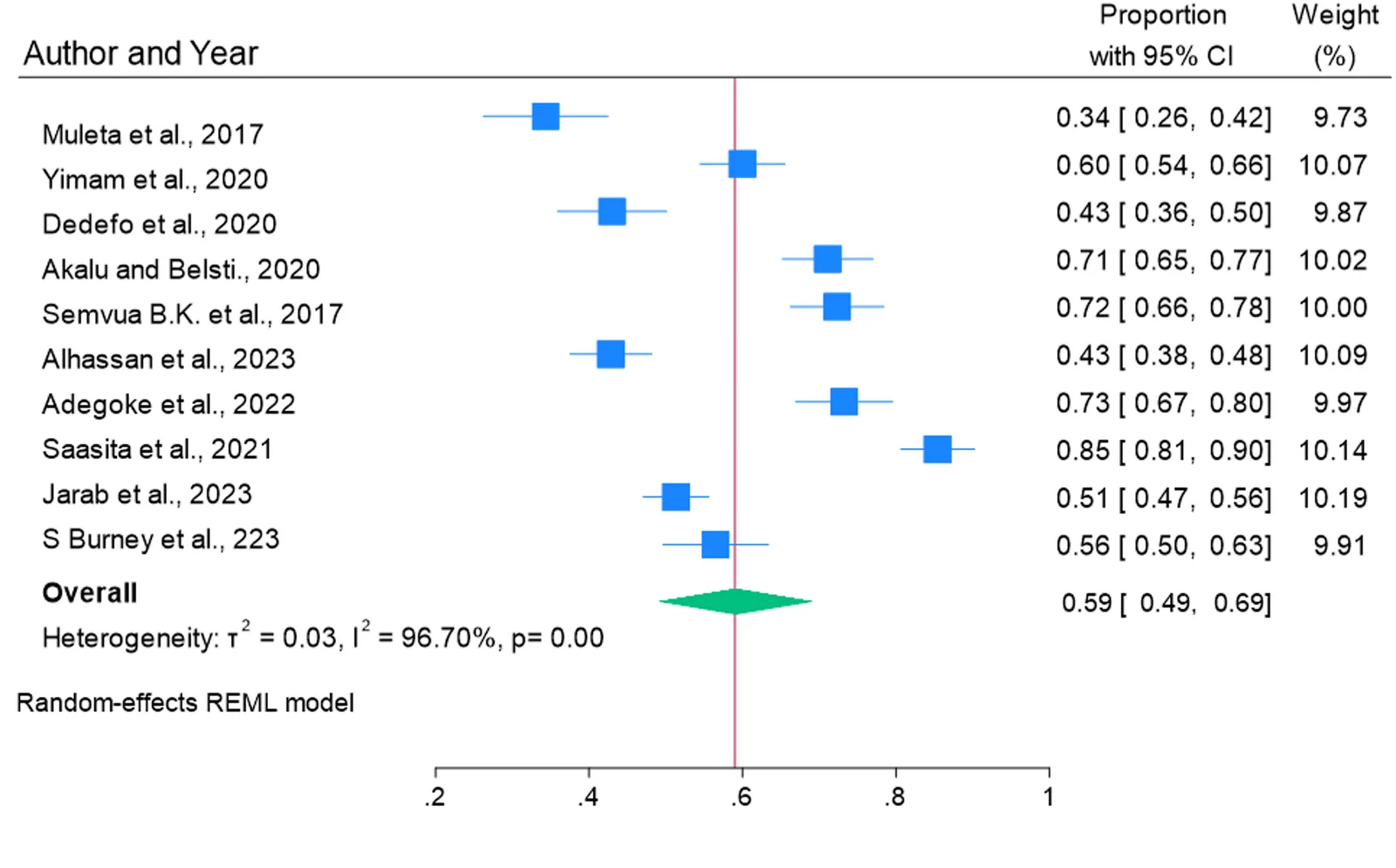
The forest plot shows the pooled prevalence of patients with poor glycemic control in diabetic hypertensives based on FPG and HbA1c measurements

**Fig. 3:**
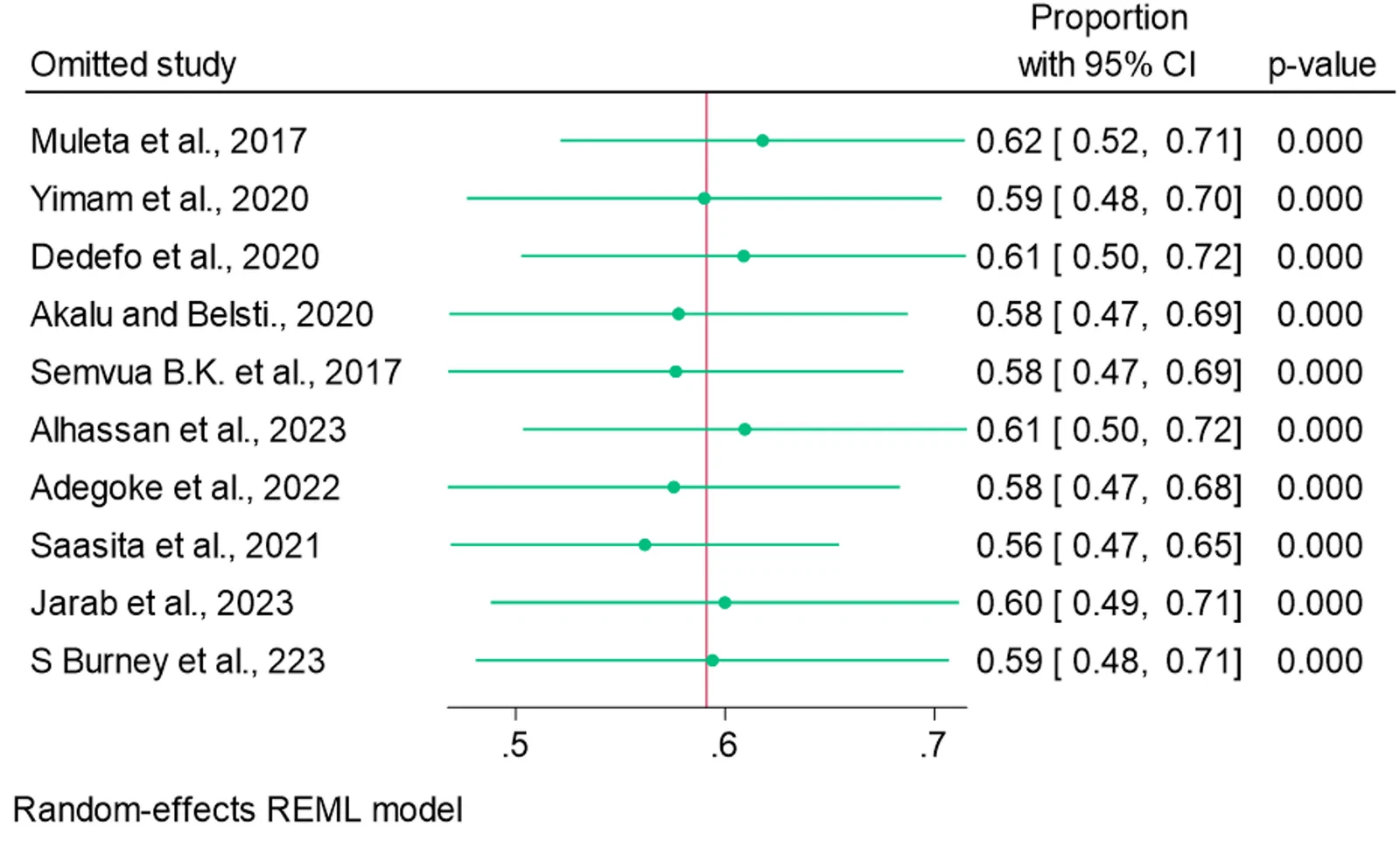
Leave-one-out sensitivity analysis

According to this meta-analysis, the pooled prevalence of poor glycemic control among diabetic hypertensive patients was 59% (95% CI: 0.49–0.69) based on FBG and HbA1c measurements (FBG > 130 mg/dl and HbA1c ≥ 7) using the random effect model (Show in Fig. 2). Heterogeneity (I^2^ = 96.70%, p-value = 0.00) as indicated in the forest plot. The individual pooled prevalence was extended from 34% to 85%. Furthermore, A sensitivity analysis was performed using a leave-one-out method to assess the robustness of the findings. The results indicated that all point estimates fell within the 95% Confidence Interval (shown in Fig. 3).

### Pooled prevalence of poor glycemic control based on diagnostic criteria

**Fig. 4:**
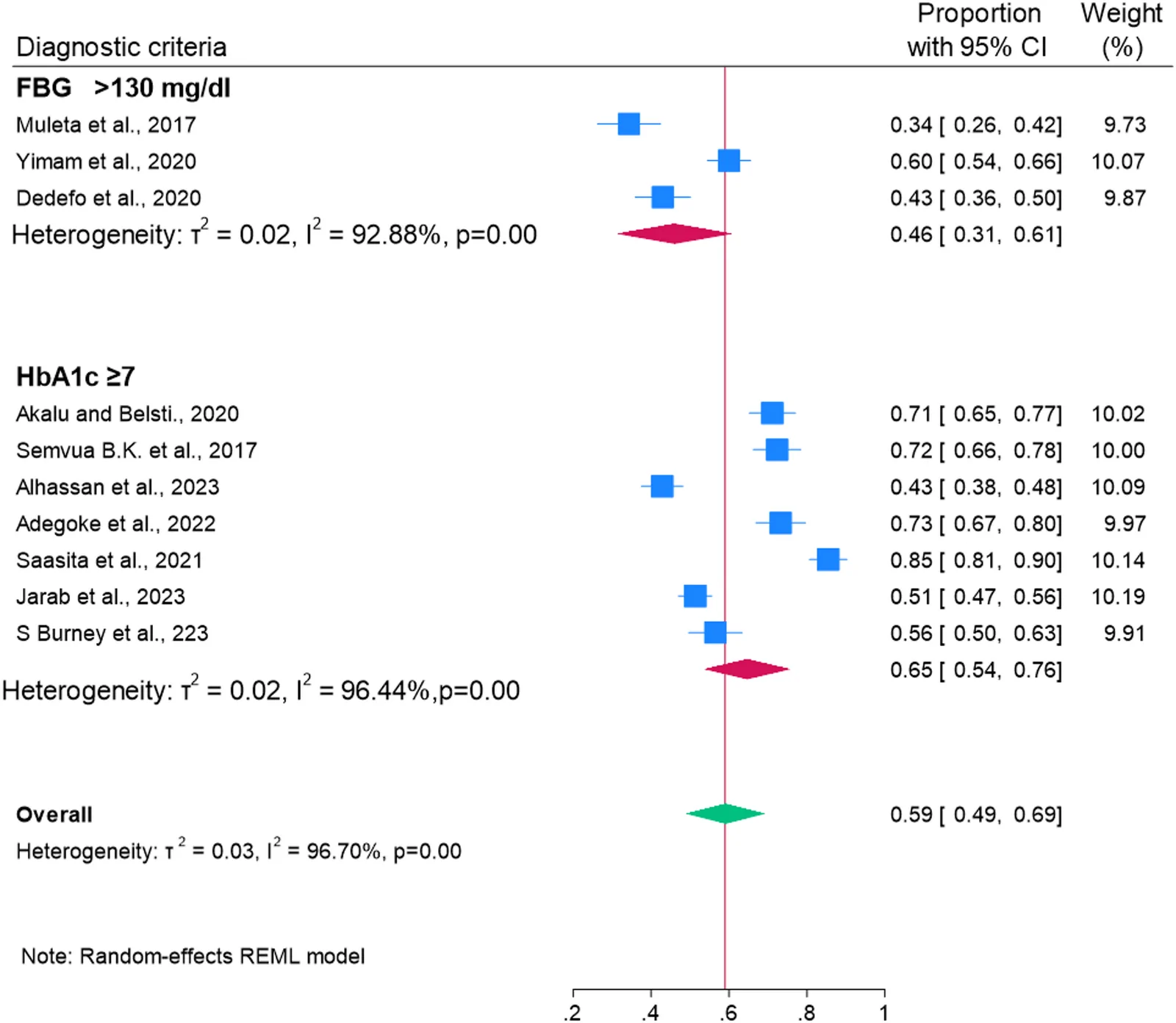
Forest plot; proportion based on diagnostic criteria of poor glycemic control in diabetic hypertensive patients based on FBG and HbA1c measurements

Based on FBG and HbA1c measurements, the pooled prevalence of poor glycemic control in diabetic hypertensive patients was 46% (95%CI: 0.31–0.61) based on FBG>130 mg/dl, and 65% (95%CI: 0.54–0.76) based on HbA1c ≥ 7%. (as indicated in Fig. 4)

### Heterogeneity

**Fig. 5:**
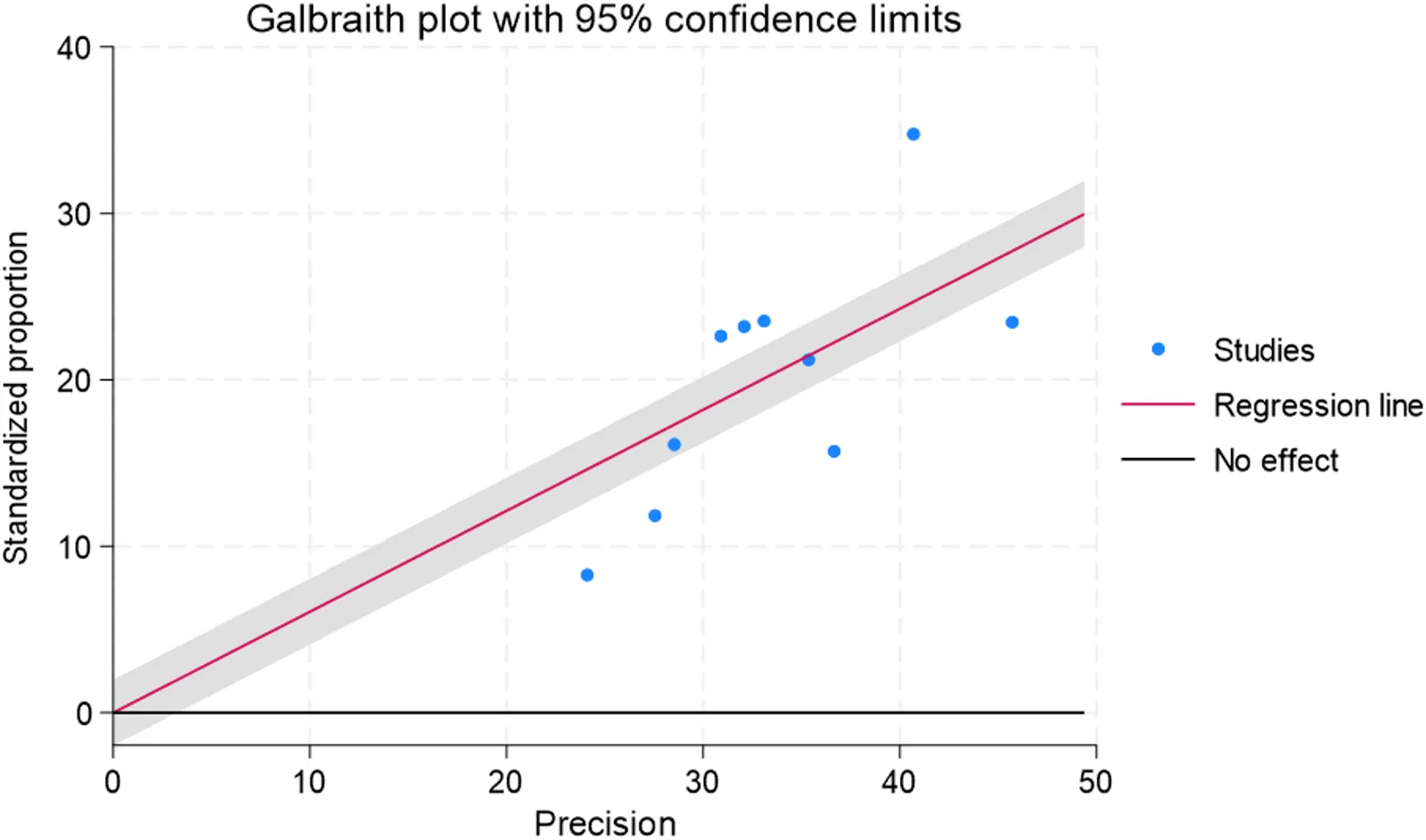
Galbraith plot: visualizes the heterogeneity for meta-analysis of the participants of diabetic hypertensive patients

A Galbraith plot indicates a positive relationship between precision and effect size. The studies indicate the possibility of publication bias in the overall analysis (as indicated in Fig. 5).

### Publication bias

**Fig. 6:**
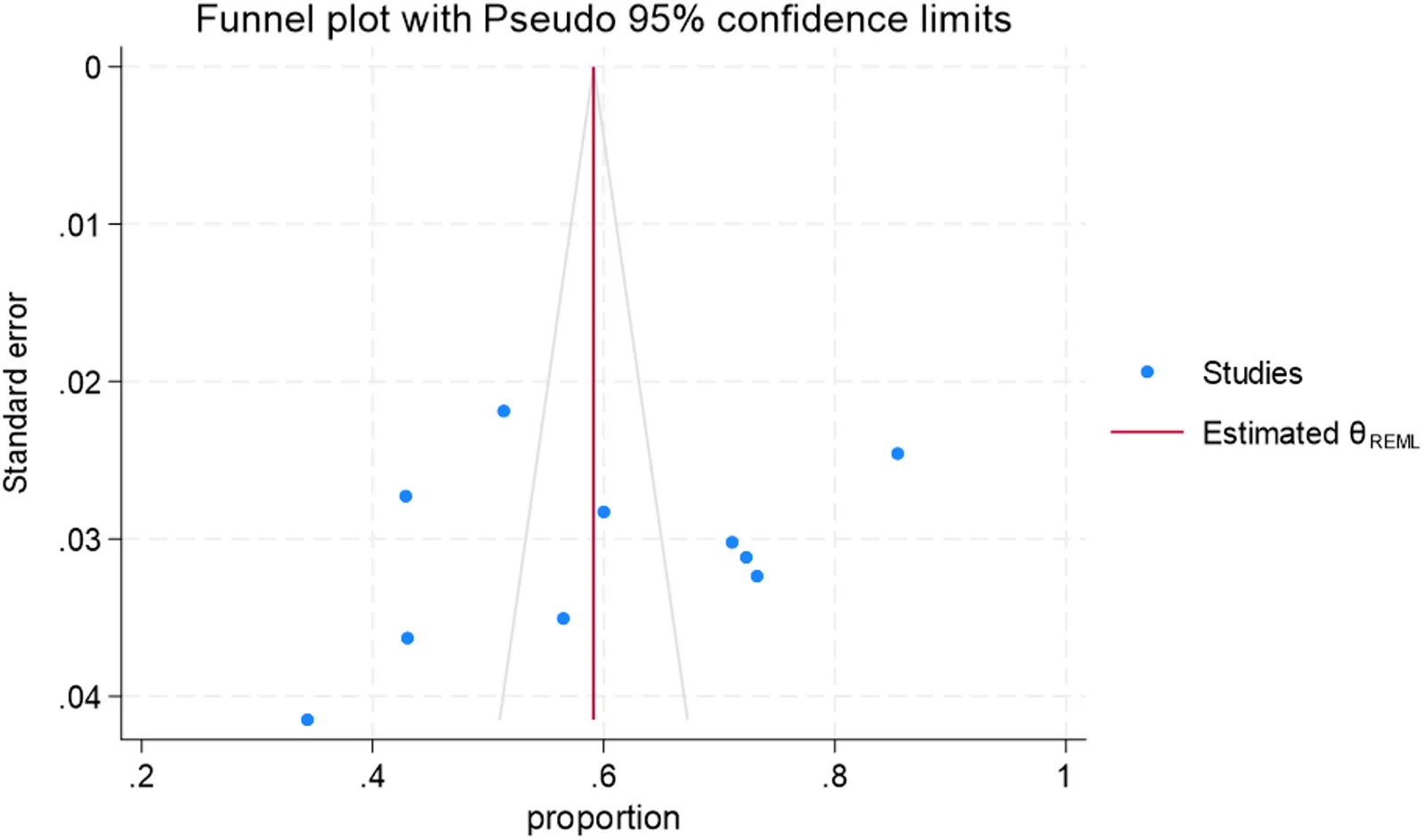
Funnel plot; visual inspection of publication bias for meta-analysis of studies of participants with diabetic hypertensive patients

In meta-analyses, visual inspection of the funnel plot indicated that publication bias cannot be entirely ruled out. However, no statistically significant publication bias was detected (as indicated in Fig. 6).

## Discussion

This systematic review and meta-analysis were conducted primarily to determine the overall estimates of poor glycemic control in diabetic hypertensive patients. Glycemic control was assessed using two parameters: FBG and A1C. According to this finding, nearly two-third of the study participants, 59% of the diabetic hypertensive patients, have poor glycemic control. This study is consistent with a previous meta-analysis conducted in Ethiopia on T2DM (61.11%) [35], the Middle East and North Africa reported T2DM (63%) [36], and a meta-analysis conducted in Ethiopia to determine poor glycemic control (61.92%) [14]. Our finding was higher than that of a study conducted in sub-Saharan Africa among DM (30.3%) [37]. This finding was lower than the systematic review and meta-analysis conducted in Ethiopia on DM (65.6%) [38], as well as individual studies conducted in Bangladesh (82%) [39], and Tanzania (69.7%) [40].

This discrepancy in the findings could be attributed to differences in glucose testing methods, cutoff points, environmental factors, and lifestyle, all of which predispose individuals to various risk factors for poor glycemic control in the study. It is also due to fragile health-care systems, a lack of community engagement in health-improvement efforts, complex treatment regimens, polypharmacy (adherence issues), longer diabetes duration, and a lack of information about glycemic control in low- and middle-income countries [37, 41]. The possible explanation for suboptimal glycemic control is associated with an increased risk of hypertension in individuals with diabetes, as well as a significant correlation between low educational attainment and cardiometabolic comorbidities, along with the progression of chronic degenerative diseases [3, 42, 43]. Multiple mechanisms may account for the association between glycemic control and hypertension. Elevated HbA1c levels may indicate impaired pancreatic β-cell function and insulin resistance. Furthermore, high HbA1c concentrations have been associated with proinflammatory cellular signaling and oxidative stress, which may contribute to arterial stiffness [44, 45].

According to the measurement, the pooled prevalence of poor glycemic control in patients with diabetes and hypertension was 46% for FBG > 130 mg/dl and 65% for HbA1c ≥ 7%. This finding was lower than the meta-analysis conducted in DM (66.8% and 78.2%) [38]. High HbA1c levels may regulate blood lipids, increasing blood viscosity and the risk of cardiovascular illnesses. Poor glycemic control levels increase macrovascular and microvascular complications and are associated with a higher risk of hypertension among individuals with diabetes [46]. The coexistence of diabetes and hypertension accelerates the advancement of diabetes-related problems and correlates with an increased risk of cardiovascular mortality. The possible explanation may be that insufficient physical activity is significantly correlated with poor glycemic control. A comprehensive multicenter study involving over 18,000 diabetic patients indicated that physical activity is inversely related to HbA1c, diabetic ketoacidosis, diabetes-associated comorbidities, body mass index, dyslipidemia, and hypertension [47, 48].

Based on the evidence presented in the literature, depression is likely an independent risk factor for hypertension, and diabetic patients may exhibit a decline in quality of life, diminished self-care capabilities, poor glycemic control, and an increased risk of macrovascular and microvascular complications [49]. Lifestyle interventions significantly reduced glycosylated hemoglobin (HbA1c) levels compared to standard therapy for patients with type 2 diabetes mellitus [50]. Appropriate therapies, educational programs, exercise programs, increasing universal health coverage, and adherence to dietary recommendations are essential to maintain good glucose control and prevent the development of life-threatening complications [50, 51].

The pooled estimates showed substantial heterogeneity. Although a subgroup analysis was conducted to identify potential sources of variability, heterogeneity remained significant and unexplained. This variability may be due to differences in study demographics, outcome measurement, and healthcare settings. Also, the variability may be due to comorbidity burden, adherence patterns, and study setting. So, these findings may be interpreted with caution. This review suggests that policymakers emphasize the importance of modernizing healthcare systems in developing countries, implementing multifaceted therapies to improve glucose control, and optimizing lifestyle, all of which are essential for management. This limitation might affect the extent to which our findings can be applied and should be taken into account when interpreting the pooled estimates.

## Strengths and limitations of the study

This systematic review and meta-analysis possess several notable strengths. The study was conducted in accordance with PRISMA guidelines and was prospectively registered with PROSPERO. The JBI critical appraisal tools were employed to evaluate methodological quality, and pooled prevalence estimates were computed using random-effects models. Sensitivity analysis and publication bias assessments were performed to improve the robustness of the findings. There are some limitations. The pooled estimates indicated substantial heterogeneity; however, it remained unexplained. These differences may be attributable to variations in demographic characteristics and measurement instruments. Most primary studies were conducted in high-income nations, thereby limiting the applicability of the findings to the global population. Restricting to English-only could potentially cause selection bias. We excluded studies that solely focused on diabetes, hypertension, and other chronic conditions, except for diabetic-hypertensive patients. Given these constraints, the results should be interpreted with caution.

## Conclusion

This systematic review and meta-analysis show that poor glycemic control is highly prevalent in patients with coexisting diabetes mellitus-hypertension, with an overall pooled prevalence of 59%. When evaluated against diagnostic criteria, nearly half of the participants assessed with FPG and two-thirds of those assessed with HbA1C exhibited inadequate glycemic control. However, given the significant heterogeneity and variability in diagnostic methods, these findings may be interpreted with caution. This finding is important as a baseline for refining clinical guidelines and improving long-term outcomes for this high-risk group. Future researchers will evaluate effective interventions to improve glycemic control in diabetic hypertensive patients across diverse study settings.

## Supplementary Information

Below is the link to the electronic supplementary material.

Supplementary Material 1

## Data Availability

All data analyzed in this study were included in this published article.

## Abbreviations

A1C: Glycated hemoglobin
CVDs: Cardiovascular diseases
DM: Diabetes mellitus
FBG: Fasting blood glucose
HTN: Hypertension
T2DM: Type 2 diabetes mellitus
